# Clinical application of computed tomography-guided ^125^I seed interstitial implantation for head and neck cancer patients with unmanageable cervical lymph node metastases

**DOI:** 10.1186/s40001-016-0213-1

**Published:** 2016-04-27

**Authors:** Hai Huang, Shaonian Xu, Fusheng Li, Zhenguang Du, Liang Wang

**Affiliations:** Department of Orthopedic Oncology, The People’s Hospital of Liaoning Province, Wenyi Street No. 33, Shenyang, 110016 China

**Keywords:** Cervical lymph node metastasis, Head and neck cancer, CT-guided ^125^I seed, Interstitial implantation

## Abstract

**Background:**

To assess clinical application of computed tomography (CT)-guided ^125^I seed implantation for patients who cannot endure or unwillingly receive repeated surgery, chemotherapy, or radiotherapy for unmanageable cervical lymph node metastases in head and neck cancer (HNC).

**Methods:**

Thirty-one consecutive patients received CT-guided ^125^I seed implantation between February 2010 and December 2013. To evaluate the clinical efficiency, karnofsky performance score (KPS), numeric rating scale (NRS), and tumor volume at 3-, and 6-month post-implantation were compared with pre-implantation, along with local control rate (LCR), overall survival rate (OSR), and complications at 3, 6 months, 1, and 2 years.

**Results:**

The tumor volume was obviously decreased at 3-, and 6-month post-implantation (21.23 ± 8.83 versus 9.19 ± 7.52 cm^2^; 21.23 ± 8.83 versus 6.42 ± 9.79 cm^2^; *P* < 0.05) compared with pre-implantation. The NRS was statistically reduced (3.06 ± 1.06 versus 7.77 ± 0.92; 2.39 ± 1.15 versus 7.77 ± 0.92; *P* < 0.05), while KPS was significantly improved (83.18 ± 5.97 versus 73.60 ± 7.90; 82.86 ± 5.43 versus 73.60 ± 7.90; *P* < 0.05) postoperatively at 3 and 6 months, respectively. The LCR at 3, 6 months, 1, and 2 years was 96.30, 83.87, 64.51, and 45.16 %, respectively. The OSR was 100, 100, 67.74, and 45.16 %, respectively. Three cases experienced grade I and two had grade II acute radiation toxicity.

**Conclusions:**

CT-guided seed implantation may be feasible and safe for HNC patients whose neck nodes are not manageable by routine strategies with fewer complications, higher LCR, and significant pain relief.

## Background

Head and neck cancer (HNC) is a broad term encompassing epithelial malignancies, which is the sixth most common type of cancer in the world [1]. The incidence of this disease is increasing worldwide [2, 3]. Although advances in multimodality therapies (for example, surgery combined with adjuvant external-beam radiotherapy (EBRT), or adjuvant radiochemotherapy) for patients with HNC, the clinical outcomes are still poor [4]. The 5-year survival rate remains relatively unchanged for the past 30 years [5, 6]. Cervical lymph node metastasis might contribute to this bleak scenario [7], because HNC has a high propensity to metastasize to locoregional lymph nodes due to the rich lymphatic system [8]. Moreover, the presence of cervical lymph node metastasis is now considered as the single most adverse independent prognostic factor [8–12], and the status of lymph node involvement decreases the overall survival rate (OSR) by nearly 50 % [13, 14]. Therefore, it is necessary to search an effective therapeutic regimen.

In recent years, computed tomography (CT)-guided ^125^I seed implantation has received much attention in the field of medical science. It is a minimally invasive brachytherapy technique that ensures protracted cell killing over a period of several months through targeted delivery of high-dose radiation. Accumulating evidence suggests that this technique is considered as an important supplementary treatment for unresectable advanced HNC due to its ability to offer high precision, little invasiveness, strong lethality, and less complications [4, 15–18]. Moreover, these studies have shown that this technique offered significant improvement in clinical symptoms and life quality.

However, rare clinical data on the treatment of patients with cervical lymph node metastases in HNC have rarely been reported. In the present study, CT-guided ^125^I seed implantation was performed to patients with cervical lymph node metastases in HNC. The results identified by this study might contribute to a better evidence of treatment for HNC patients with unmanageable cervical lymph node metastases.

## Methods

### Subjects

This study was approved by The People’s Hospital of Liaoning Province Ethics Committee, and informed consent was obtained from all the enrolled patients. Between February 2010 and December 2013, 31 consecutive patients with unmanageable cervical lymph node metastases in HNC, including 19 men and 12 women, aged 48–75 years (mean 67 years), who received CT-guided ^125^I seed interstitial implantation in our hospital were included in this study. The source of the metastatic tumor included nine cases of nasopharyngeal carcinoma, eight cases of tongue cancer, five cases of laryngeal carcinoma, three cases of maxillary sinus carcinoma, and six cases of thyroid carcinoma. The previous surgeries performed on the patients included 18 cases of radical neck dissection (RND) and 13 cases of selective or modified neck dissection (MND). The primary tumor stage was all stage IV for all patients. The tumor-node-metastasis (TNM) staging included three cases of T3N3M1, 11 cases of T2N3M1, four cases of T2N2M1, six cases of T2N1M1, two cases of T3N1M1, one case of T1N1M1, one case of T1N3M1, one case of T4N3M1, one case of T1N2M1, and one case of T3N2M1. Among the 31 patients, 25 patients underwent one surgery, while six patients underwent surgery twice. All patients had undergone prior adjuvant external-beam radiation therapy (EBRT). Of these patients, 25 patients underwent EBRT once, and six patients underwent EBRT twice. The cumulative radiotherapy dose was 28-90 GY (mean 66 ± 13.34 GY). In addition, six patients received chemotherapy for four cycles and eight patients accepted chemotherapy for three cycles (Table 1).

**Table 1:** General characteristics of 31 patients

Patient no.	Age	Gender	Location of primary tumor	TNM classification	Site of metastases	Pre-procedural NRS	Post-procedural NRS (6 months)	Size of lymph node metastasis (cm*cm)	Pre-treatments
1	59	M	NPC	IVT3N3M1	L	8	3	8*5	Cisplatin + fluorouracil (4), radiotherapy (2)
2	63	M	NPC	IVT1N1M1	L	7	2	3*3	Cisplatin + fluorouracil (4)
3	65	F	NPC	IVT2N3M1	L	7	2	6*3	Cisplatin + fluorouracil (4)
4	60	M	NPC	IVT2N2M1	L	7	2	4*3	Cisplatin + fluorouracil (4), radiotherapy (2), surgery (2)
5	55	F	NPC	IVT3N3M1	R	9	2	7*4	
6	61	M	NPC	IVT2N1M1	R	9	3	5*2	Cisplatin + fluorouracil (3)
7	63	F	NPC	IVT2N1M1	L	8	3	5*4	Cisplatin + fluorouracil (3)
8	54	F	NPC	IVT1N3M1	R	7	3	6*3	Radiotherapy (2)
9	52	M	NPC	IVT4N3M1	L	9	2	8*5	Cisplatin + fluorouracil (4) surgery (2)
10	70	M	Tongue cancer	IVT1N2M1	R	8	2	3*3	
11	65	M	Tongue cancer	IVT2N3M1	L	7	3	5*3	Cisplatin vincristine methotrexate (3)
12	72	F	Tongue cancer	IVT2N2M1	L	8	2	5*4	
13	68	M	Tongue cancer	IVT2N3M1	R	6	2	4*4	Cisplatin vincristine methotrexate (3)
14	74	M	Tongue cancer	IVT2N3M1	L	8	2	5*4	Radiotherapy (2)
15	71	F	Tongue cancer	IVT2N3M1	R	7	1	4*3	
16	69	F	Tongue cancer	IVT2N3M1	R	7	2	4*4	Cisplatin vincristine methotrexate (3)
17	67	F	Tongue cancer	IVT3N3M1	L	9	3	6*7	Cisplatin vincristine methotrexate (3)
18	75	M	Laryngocarcinoma	IVT2N3M1	R	9	2	5*4	Surgery (2)
19	69	M	Laryngocarcinoma	IVT3N2M1	R	8	3	7*4	
20	70	M	Laryngocarcinoma	IVT2N3M1	R	9	2	5*3	Radiotherapy (2)
21	73	M	Laryngocarcinoma	IVT2N3M1	R	8	2	6*5	
22	70	F	Laryngocarcinoma	IVT2N2M1	R	9	2	4*4	Surgery (2)
23	75	M	Maxillary sinus carcinoma	IVT2N3M1	R	8	2	6*4	Carboplatin (4)
24	70	M	Maxillary sinus carcinoma	IVT2N2M1	R	7	2	5*4	Cisplatin (3)
25	51	F	Maxillary sinus carcinoma	IVT2N3M1	L	6	2	6*4	Cisplatin (3)
26	75	F	Thyroid cancer	IVT2N1M1	R	7	8	6*5	Radiotherapy (2)
27	57	M	Thyroid cancer	IVT2N1M1	L	8	2	5*5	
28	61	M	Thyroid cancer	IVT3N1M1	R	9	2	5*3	
29	59	F	Thyroid cancer	IVT2N1M1	L	7	2	4*4	Surgery (2)
30	48	M	Thyroid cancer	IVT3N1M1	L	7	2	5*4	
31	74	M	Thyroid cancer	IVT2N1M1	L	8	2	6*5	Surgery (2)

*M* male, *F* female, *L* left cervical lymph node, *R* right cervical lymph node, *NPC* nasal pharyngeal cancer

The indication for CT-guided ^125^I seed implantation was as follows: (1) clinically diagnosed as cervical lymph node metastasis, with HNC being confirmed by pathology; (2) Karnofsky performance score (KPS) ≥ 60; (3) normal function in liver, kidneys, and heart; (4) persistent pain of cervical tumors and treatment of medication, physical therapy, or other conservative therapy made no significant improvement; (5) expected lifetime was more than 3 months; (6) the trachea, esophagus or other vital organs were compressed by cervical metastatic tumor, which had seriously affected the quality of the patient’s life; and (7) patients were not suitable for reoperation, chemotherapy, or radiotherapy, or the patients were not willing to receive any chemotherapy or radiotherapy.

### Equipment and CT-guided implantation

**Fig. 1:**
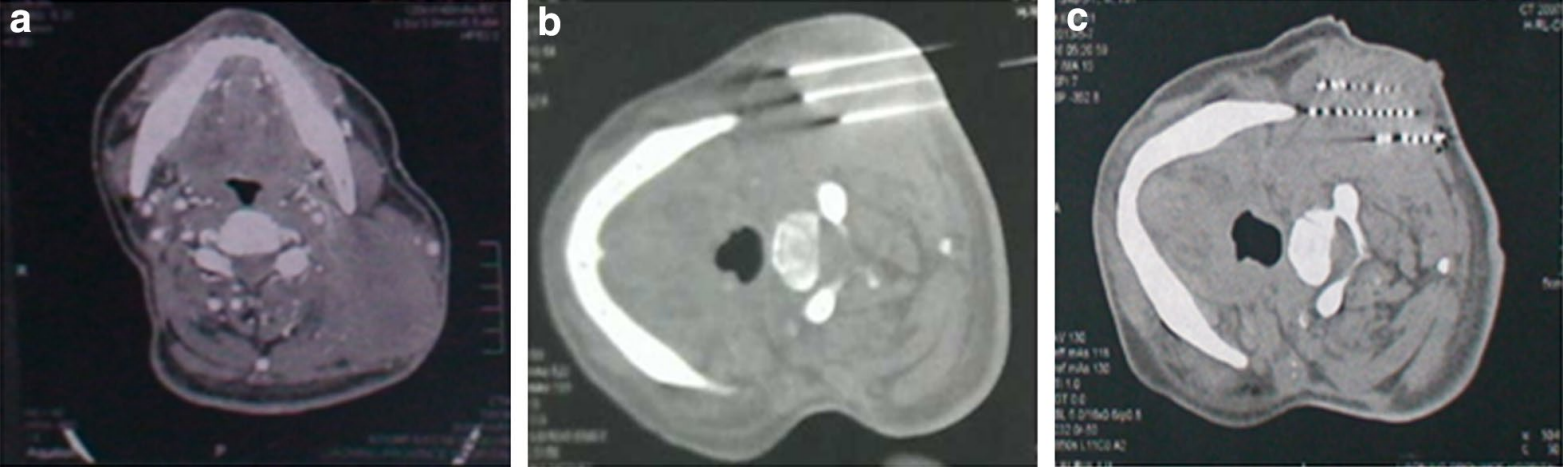
Treatment of a patient with cervical lymph node metastasis in primary nasopharyngeal carcinoma. **a** CT scan shows left neck lymph node metastasis before implantation. **b** CT scan shows that ^125^I seeds are implanted into the tumor via implantation needles. **c** CT scan shows that ^125^I seeds are implanted post-implantation 3 months. *CT* computed tomography

Before the operation, the history and physical condition, hematological and chemical profiles of all the patients were evaluated. Additionally, each patient underwent a detailed tumor volume study using contrast-enhanced CT scanning (GE Lightspeed 64, General Electric Company, Fairfield, CT, USA, thickness = 5 mm) of the neck 1 week before seed implantation (Fig. 1a). The planning target volume (PTV) was extended beyond the clinical target volume (CTV) by a 1-cm margin, which was outlined by the radiation oncologist on each transverse image. Treatment Planning System (TPS, Prowess 3D, SSGI, Chico, CA, USA) was performed to calculate the dose distribution, which was based on the American Association of Physicists in Medicine TG43 brachytherapy formalism [19]. The ^125^I seed sources were purchased from ZhiBo Carve Out Development Ltd. Beijing, China. It releases rays with a nominal activity of 0.4–0.8 mCi/seed, a half-life of 60.2 days, and a size of 0.8 × 0.45 mm. The 18-G implantation needles and turntable implantation gun (ZhiBo Carve out Development Ltd. Beijing, China) were utilized for seed implantation. The depth and angle of implantation needles were guided under CT supervision, avoiding important vessels and nerves. Patients were allowed a clear liquid diet for 24 h before the implantation. This procedure was carried out in a standard CT room under local anesthesia by using 0.5 % lidocaine. The distance between the two adjacent implantation needles was about 1 cm (Fig. 1b). Repeated CT was performed within 24 h after the implantation to confirm the distribution of seeds, and then the images were collected and put into the TPS (Fig. 1c). Re-implantation was performed if necessary. After the implantation, all patients were kept under observation, and then returned toward only if there was no sign or symptom of complications, such as bleeding. Then, anti-inflammatory and hemostasis were given to all the subjects after implantation.

### Effect evaluation

Tumor response was evaluated according to the World Health Organization (WHO) standard criteria [20]. Tumor size was estimated from bidimensional measurements (product of the longest diameter and its longest perpendicular diameter for each tumor). CT examination was conducted at 3 and 6 months after the implantation and classified as follows: a complete response (CR) indicated the disappearance of all tumoral lesions lasting for more than 4 weeks; partial response (PR) indicated that total tumor size decreased at least a 50 %; stable disease (SD) indicated that total tumor size decreased by less than 50 % or increased by less than 25 %; and progressive disease (PD) indicated an exacerbation of tumor size at least a 25 % or lager.

In the present study, local control rate (LCR) was defined as the absence of tumor progression in CT (SD + PR + CR), while overall survival rate (OSR) was defined as the percentage of patients who are alive after a specified number of years of follow-up from the start point of post-implication to the end of follow-up or death.

The numerical rating scale (NRS) was used to measure of pain intensity before and after operation [21]. The NRS is an 11-point scale, which consists of integers from 0 through 10 where 0 represents no pain; 1–3, mild pain without affecting sleep; 4–6, moderate pain; 7–9, severe pain (affect sleep or wake up from the pain); and 10 represents the worst imaginable pain. Patients were inquired for the pain degree and were required to select a single number during 0–11 that could best reveal their pain intensity and this selected number was the NRS.

Furthermore, a Radiation Therapy Oncology Group (RTOG) was utilized to assess the acute radiation morbidity, and the RTOG/European Organization for Research and Treatment of Cancer (RTOG/EORTC) was employed to assess the latest radiation morbidity [22].

### Follow-up

All patients received follow-up phase immediately after the implantation. The follow-up period was 6–38 months, with first visits at the third month after intervention, and then subsequent follow-ups occurred at the 6 months, 1, and 2 years. The tumor volume was recorded at 3- and 6-month post-implantation using CT images. The LCR and OSR at 3, 6 months, 1 and 2 years were assessed. All patients successfully completed the follow-up.

### Statistical analysis

All data were expressed as mean ± SD, and the statistical analysis was performed by using the SPSS software version 19.0 (SPSS, Chicago, IL, USA). The median survival time of survival analysis was assessed by Kaplan–Meier methods. The difference of pain relief before and after treatment was analyzed by paired *t* test. *P* < 0.05 was considered statistically significant.

## Results

### Seed implantation characteristics

The seed implantation procedure was successfully conducted in all patients. The implanted number of ^125^I seeds was 16–84 (mean 46.13 ± 16.28) for each patient. The mean activity of each seed was 0.4–0.8 mCi (mean 0.65 ± 0.15). The D90 of ^125^I seeds ranged from 90–125 Gy (mean 101.03 ± 8.54 Gy).

### Response to treatment

**Fig. 2:**
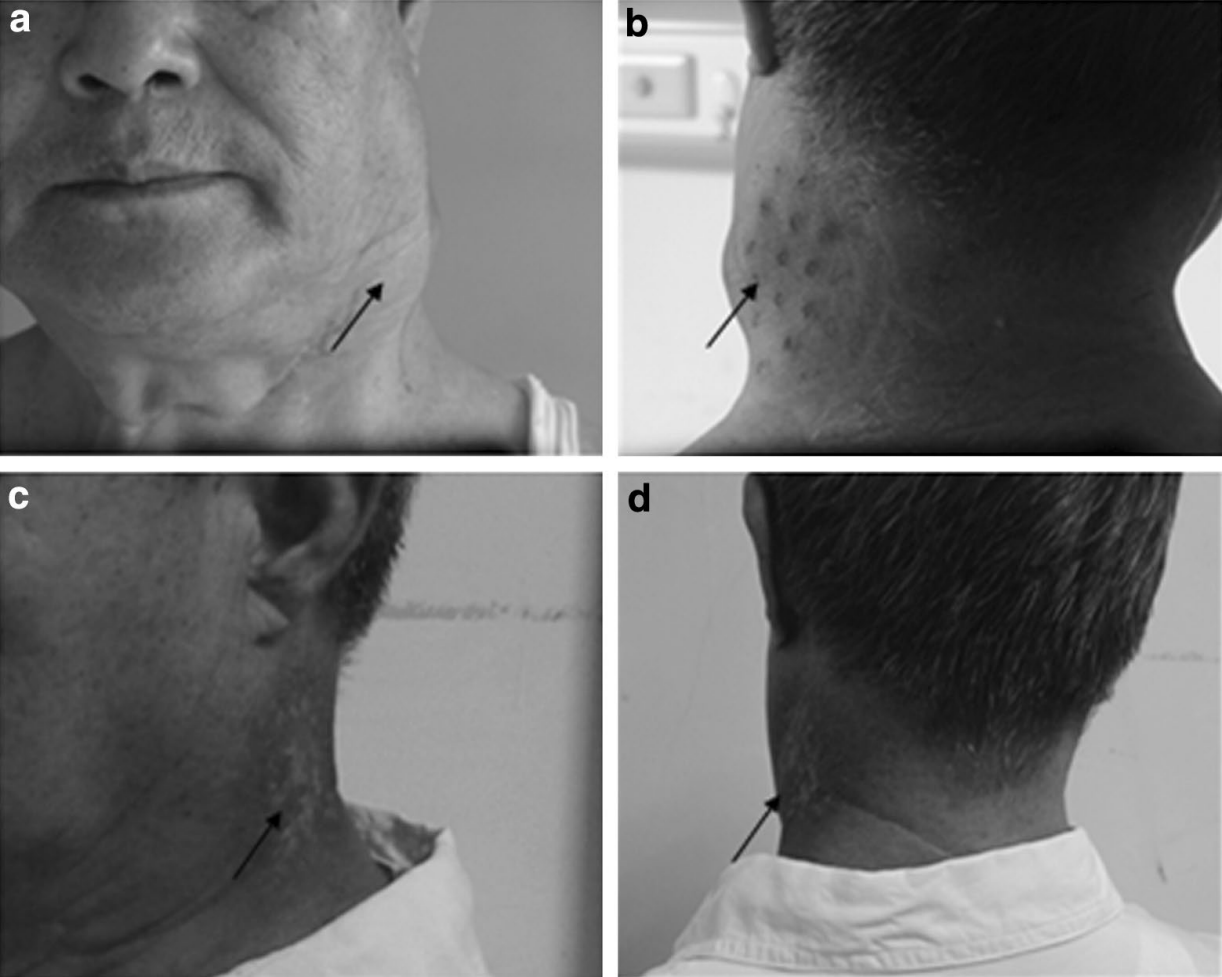
Pre-implantation and post-implantation photos of the patient. **a**–**c** Masses in the neck of the patient before implantation. Neck masses were pointed by *arrowheads*. **d** Post-implantation photo shows that the neck masses are significantly reduced at 3 months after implantation

**Fig. 3:**
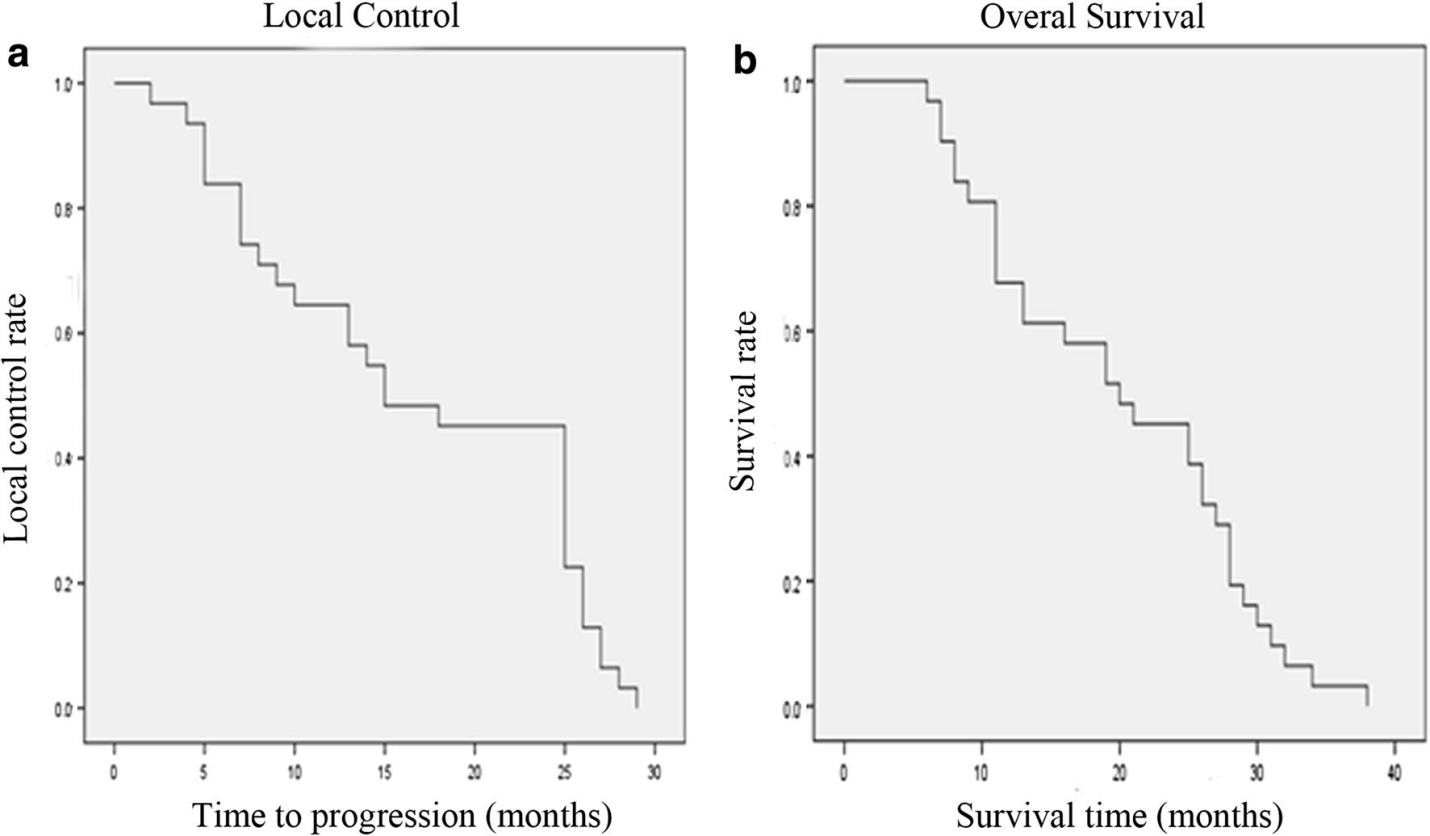
Local control curve and overall survival curve after CT-guided ^125^I implantation. **a** Local control curve after CT-guided ^125^I implantation. **b** Overall survival curve after CT-guided ^125^I implantation. *CT* computed tomography; Local control rate (LCR) was defined as the absence of tumor progression in CT (stable disease + partial response + complete response); Overall survival rate (OSR) was defined as the percentage of patients who are alive after a specified number of years of follow-up from the start point of post-implication to the end of follow-up or death

After ^125^I seed interstitial implantation, the tumor volume was significantly reduced from 21.23 ± 8.83 cm^2^ pre-implantation to 9.19 ± 7.52 and 6.42 ± 9.79 cm^2^ at 3 months and 6 months after implantation (*P* < 0.05) (Table 2; Fig. 2). Repeated CT conducted at 3-month post-implantation showed that there were three cases (9.68 %) in CR, 25 cases (80.65 %) in PR, two cases (6.45 %) in SD, and one case (3.22 %) in PD, respectively. The NRS scores for pre-implantation, and 3- and 6-month post-implantation were 7.77 ± 0.92, 3.06 ± 1.06, and 2.39 ± 1.15, respectively. Symptoms of pain were significantly reduced postoperatively at 3 and 6 months (*P* < 0.05). Furthermore, KPS significantly improved at 3- and 6-month post-implantation compared with pre-implantation (83.18 ± 5.97 versus 73.60 ± 7.90; 82.86 ± 5.43 versus 73.60 ± 7.90; *P* < 0.05), respectively. As shown in Fig. 3, the LCR at 3, 6 months, 1 and 2-years was 96.30, 83.87, 64.51, and 45.16 %, respectively. The OSR was 100, 100, 67.74, and 45.16 %, respectively. Four patients died of local recurrence; 11 patients died of lung metastasis; 13 patients died of recurrent tumor and distant metastases; and 3 patients had severe heart disease and died of heart disease, which was unrelated to the tumor.

**Table 2:** Effect evaluation of CT-guided ^125^I seed implantation

	Pre-implantation	Three months post-implantation	Six months post-implantation
Tumor volume (cm^2^)	21.23 ± 8.83	9.19 ± 7.52^#^	6.42 ± 9.79^#^
NRS	7.77 ± 0.92	3.06 ± 1.06^#^	2.39 ± 1.15^#^
KPS	73.60 ± 7.90	83.18 ± 5.97^#^	82.86 ± 5.43^#^

*KPS* Karnofsky performance score, *NRS* Numerical rating scale

Compared with pre-implantation, ^#^ *P* < 0.05

### Complications

Of all the patients, three patients suffered from fever post-implantation, but the fever did not exceeded 38.5 °C, and body temperature returned to normal in 3–5 days. Additionally, 3 and 2 patients developed grade I and grade II acute radiation toxicity on the skin (for example, faint or dull erythema/epilation/dry desquamation/moderate edema) and mucous membrane (for example, mild/moderate pain, patchy mucositis), respectively. Moreover, 1, 2, and 1 patients experienced grade I, grade II, and late radiation toxicity on the skin (pigmentation change), subcutaneous tissue (for example, slight in duration and loss of subcutaneous fat) and mucous membrane (dryness), respectively. Other tissues, such as the eyes, spinal cord, and lung, were not involved. No patients developed grade III, IV, or V acute/late radiation toxicity, or other serious complications (for example, massive hemorrhage, acute pulmonary embolism, or fistula).

## Discussion

In the present study, 31 patients with unmanageable cervical lymph node metastases in HNC who could not undergo or not be willing to receive repeated surgery, chemotherapy, and radiotherapy (for example, EBRT) were enrolled. CT-guided ^125^I seed implantation was successfully performed to all patients. The clinical application of this technique was evaluated. The results showed that tumor volume and NRS scores were significantly reduced and KPS was significantly improved at 3- and 6-month post-implantation compared with pre-implantation. Additionally, fewer complications and higher LCR and OSR presented post-implantation.

For patients with either clinical positive or negative neck lymph node metastasis in HNC, high-dose radiotherapy and radical surgery are the main treatments [12, 23]. However, various postoperative pains and disorders might be induced [24], as well as radiation-associated normal tissue injury [25], even cerebrovascular disease [26, 27]. Although combined chemotherapy with radiotherapy has been increasing, tumor control and survival remain disappointing [28]. Up to now, effective treatments for this disease still remain challenging. Fortunately, ^125^I radioactive seeds, which are considered as an effectively salvage or palliative treatment, have received increasing attention in the recent years. It has been successfully applied to the treatment for various malignant tumors [29–31]. The major advantages of this technology are its high dose of irradiation to the target area and low dose of irradiation to the surrounding normal tissue. Besides, this technology can maximize local control and minimize morbidity.

Recent studies have also been performed to present the clinical application of ^125^I seed implantation. Goffinet et al. [32] reported that the majority of patients with advanced recurrent HNC in his study had received prior to treatment before ^125^I seed implantation. The results showed that a 70 % LCR was achieved. Park et al. [33] also reported that 35 patients with advanced recurrent squamous cell cancers of HNC received adjuvant ^125^I seed implants after surgical resection. The 5-year disease-free survival was 41 %. A study conducted by Zhu et al. assessed the feasibility, efficacy, and morbidity of ^125^I seed implantation for recurrent HNC after surgery and EBRT [16]. The results showed that the 1-, 2- and 3-year LCR were 73.3, 27.5, and 27.5 %, respectively, whereas the 1-, 2-, and 3 year OSR were 53.0, 18.2, and 18.2 %, respectively. Besides, a retrospective study on 14 patients with recurrent HNC underwent ^125^I seed implantation [4]. The results demonstrated that the 1-, 2-, 3-, and 5-year LCR were 52, 39, 39, and 39 %, respectively, and the 1-, 2-, 3-, and 5-year OSR were 65, 39, 39, and 39 %, respectively. Similarly, in our study, the LCR at 3, 6 months, 1-, 2-year was 96.30, 83.87, 64.51, and 45.16 %, respectively. The OSR were 100, 100, 67.74, and 45.16 %, respectively. Our results were slightly different from the above previous studies. The main reasons were listed as follows: (1) the number of patients and prior treatment before ^125^I seed implantation in our study were different from the above studies and (2) the patients in our study were all had cervical lymph node metastasis. However, it is worth noting that the operation and chemoradiotherapy that the patients received prior to ^125^I seed implantation may not have an indirect impact on the results due to the similarity with the previous studies.

Moreover, in the present study, the NRS scores showed that the pain is significantly relieved in patients receiving ^125^I seeds implantation. This reduction in pain may be associated with the following factors: (1) tumor cells were killed, leading to the reduction of tumor volume, therefore the tension of tumor capsule declined and the compression of adjacent organs or peripheral nerve relieved; (2) the expression of pain-related cytokines (e.g., 5-hydroxytryptamine, bradykinin, and prostaglandin) was reduced; (3) the permeation of pain-related cytokines would be blocked by fibrosis or micro-vascular thrombosis in the tumor or tissue adjacent to tumors; and (4) the conduction of pain would be blocked by the functional electrophysiological block or myelin degeneration of the nerve endings around tumor. Besides, KPS in our study was also significantly improved at 3- and 6-month post-implantation. The above results suggested that ^125^I seed implantation may significantly relieve postoperative pain and improve the life quality of the patients. In addition, no patients showed serious complications shortly after implantation, while local skin pigmentation had a long-term side effect. No obvious skin or mucosal ulcer was observed, and no serious adverse events such as hemorrhage and necrosis were reported. Excellent implant technology, which includes perfect protection for skin or mucosa inter-operatively, and a good distance between the seeds and the skin (approximately 1 cm), might effectively avoid radioactive damage on the skin.

## Conclusions

In conclusion, this study shows that CT-guided ^125^I seeds implantation is a minimally invasive technique in the treatment of cervical metastatic tumors. It is characterized by higher precision and LCR, being microtraumatic and well-tolerated, with fewer complications, and significant pain relief. It may provide an effective therapeutic measure in treatment of inoperable patients or the patients who are also not suitable for chemotherapy and radiotherapy with unmanageable cervical metastasis. Considering the short follow-up period involved in our study, a long-term follow-up may be needed to acquire a definite conclusion.
